# Umbrella Review on the Relationship between Vitamin D Levels and Cancer

**DOI:** 10.3390/nu16162720

**Published:** 2024-08-15

**Authors:** Matthias Schömann-Finck, Jörg Reichrath

**Affiliations:** 1 German University of Applied Sciences for Prevention and Health Management, 66123 Saarbrücken, Germany; 2 Department of Dermatology, The Saarland University Hospital, 66421 Homburg, Germany; joerg.reichrath@uks.eu

**Keywords:** cancer, umbrella review, 25(OH)D status, vitamin D, vitamin D status, pancreatic cancer, breast cancer, lung cancer, prostate cancer, colorectal carcinoma

## Abstract

Background: Cancer is a growing public health problem and cancer is linked to vitamin D via several mechanisms. Recent umbrella reviews on the extra-skeletal effects of vitamin D did not turn their attention to cancer. Accordingly, an overview of the current state of research is needed. Materials and methods: An umbrella review was conducted to provide an overview of systematic reviews on the association between vitamin D and incidence or mortality of breast cancer, colorectal cancer, lung cancer, pancreatic cancer, and prostate cancer. Results: Inverse correlations were found between the vitamin D level (measured by circulating 25(OH)D) and mortality for all five types of cancer. For breast cancer, colorectal cancer, lung cancer, and pancreatic cancer, there are also hints of a lower incidence due to higher 25(OH)D levels. Conclusion: As most reviews include observational studies, conclusions on causality cannot be made. Methodological differences between the included reviews and different study designs in the individual studies lead to methodological problems. Despite these problems, the review shows inverse correlations between 25(OH)D levels and mortality, and mostly inverse correlations between 25(OH)D levels and incidence.

## 1. Introduction

Cancer is a significant and growing public health problem, particularly in ageing western societies. In 2019, cancer was responsible for 30 percent of the mortality in western Europe, making it the second leading cause of death [1]. Given that age is one of the most important risk factors for many cancers, it is expected that the public health problem “cancer” will continue to grow. Data from the “Global Burden of Disease Project” and other sources state that there has been a 25 percent increase in global cancer mortality from 2007 to 2017. This increase is primarily attributed to the ageing populations in most developed countries [2,3,4]. Age as a risk factor for cancer is also important because older individuals are more prone to vitamin D deficiency [5,6,7,8], and numerous studies, starting with Garland and Garland in 1980 [9], have demonstrated a correlation between vitamin D deficiency and cancer incidence and mortality. Ecological studies, such as the one by Garland and Garland, have shown that higher 25(OH)D levels or increased UV B radiation (which leads to increased dermal vitamin D synthesis) have cancer protective effects. These observations are supported by findings from in vitro and in vivo studies which show several molecular mechanisms by which vitamin D regulates cellular functions implicated in cancer development [10,11,12].

Vitamin D is a fat-soluble vitamin that can be synthesized in the epidermis of the skin through the energy of ultraviolet radiation (UV-B) or can be obtained via diet or supplements. The former way is more important that the latter. The main circulating form of vitamin D, 25(OH)D (calcidiol), is hydroxylated in the liver. The measurement of 25(OH)D via blood sample is used as an approximation of the vitamin D level. However, the active form of vitamin D is 1,25(OH)2D (calcitriol), which is hydroxylated in the kidneys through a second hydroxylation. Calcitriol is also synthesized locally in different tissues. Calcitriol exerts its biological effects by binding to the nuclear vitamin D receptor (VDR). The VDR is a member of the nuclear receptor family of transcription factors. It influences target genes involved in intracellular signaling pathways, including cell growth, differentiation, adhesion, and apoptosis. These mechanisms are the links between vitamin D and cancer [10,11,13].

In 2011, Linseisen et al. [14] provided a comprehensive overview of the topic of vitamin D and cancer, with inconclusive findings ranging from possible risk reduction concerning colorectal carcinoma to no influence of vitamin D on prostate carcinoma. Since then, the research on vitamin D has grown to a “hype” [15], but recent umbrella reviews on the extra-skeletal actions of vitamin D did not turn their attention to the association between vitamin D and cancer [16,17,18]. Therefore, it seems necessary to summarize the state of evidence on the topic of vitamin D as a potentially cheap and easy-to-apply agent to counter the increasing number of cancer cases and deaths. The following umbrella review aims to provide an update on the association between 25(OH)D status/vitamin D intake and the incidence and mortality of breast, prostate, pancreatic, colorectal, and lung cancer, which are amongst the most important cancers in industrialized countries such as Germany [3,19]. In this paper, we report only the findings for the association between 25(OH)D level and cancer.

## 2. Materials and Methods

To provide an overview of the association between 25(OH)D status/vitamin D intake and the incidence and mortality of cancer, an umbrella review was conducted. Umbrella reviews are considered as an appropriate way to summarize the scientific evidence of a given topic [20,21]. A study protocol was drafted and submitted to the International Prospective Register of Systematic Reviews (PROSPERO) database (registration number CRD42021244758). The umbrella review follows the PRISMA checklist [22], which was originally developed for systematic reviews (SR). However, due to the design of this paper as an umbrella review, some of the PRISMA items have been modified. In particular, the quality assessment of studies and the presentation of results (PRISMA items 12–15). The approach in this paper (e.g., the requirements for the study types in the reviews) is based on the umbrella review by Maretzke et al., on other extraskeletal effects of vitamin D [16].

The search strategy of this review was structured by the PICOS framework [23]. The inclusion criteria were populations at risk for the aforementioned cancers or patients with these cancers. The intervention was measurement of 25(OH)D levels (and vitamin D intake, which is not reported in this paper); the outcomes in question were the incidence or mortality of the aforementioned cancers. Following the approach of Maretzke et al., systematic reviews (with or without meta-analysis) as study type were included in the umbrella review if they included at least two RCT or cohort studies as prospective study designs. Additional inclusion criteria were: studies with adults, publications in English or German, publications between 2010 and 2020.

PubMed (December 2020) and the Cochrane Library (February 2021) were searched for eligible studies. This was complemented by a hand search of reference lists of the included reviews and of the excluded narrative reviews. The search strategy in the aforementioned databases used the elements of the PICOS scheme.

The citation software Citavi (version 6.11) was used to structure the selection of the identified articles. The eligible reviews underwent thorough analysis, and the following data were extracted and transferred into separate tables for each of the five cancers: author(s), publication year, study type, investigation period, study population, exposition(s), outcome(s), calculated effect estimates, estimates for heterogeneity, subgroup analyses, included single studies, estimates for publication bias. No summary risk estimates were computed because the umbrella review included not only meta-analyses, but also reviews with qualitative statements.

The quality of the reviews that met the inclusion criteria was assessed using AMSTAR 2 (A Measurement Tool to Assess Systematic Reviews). AMSTAR 2 makes it possible to rate reviews which contain not only RCT but also observational studies, which makes it suitable for this umbrella review [24]. The tool comprises of 16 domains. Domains 2 (“Protocol registered before commencement of the review”), 4 (“Adequacy of the literature search” [at least two bibliographic databases should be searched. The report should include years and databases examined. Key words and/or MESH terms should be reported and the full search strategy available on request]), 7 (“Justification for excluding individual studies”), 9 (“Risk of bias from individual studies being included in the review” [did the review authors use a satisfactory technique for assessing the risk of bias in individual studies]), 11 (“Appropriateness of meta-analytical methods”), 13 (“Consideration of risk of bias when interpreting the results of the review”), and 15 (“Assessment of presence and likely impact of publication bias”) are considered as “critical” for the methodological quality of a review. Based on the number of weaknesses in the critical and non-critical domains, the quality of a review can be classified as “High” (no or one non-critical weakness), “Moderate” (more than one non-critical weakness), “Low” (one critical flaw with or without non-critical weaknesses), or “Critically low” (more than one critical flaw with or without non-critical weaknesses) [24]. The Oxford Centre for Evidence-Based Medicine Levels of Evidence [25] was used to rate the evidence.

## 3. Results

The literature search of PubMed and the Cochrane Library, together with the hand search, retrieved 182 articles from PubMed, 3 from the Cochrane Library, and 57 from the reference lists of the reviews that met the inclusion criteria and the excluded narrative reviews. In detail, 90 articles were on breast cancer, 52 on prostate cancer, 18 on pancreatic cancer, 69 on colorectal cancer, and 17 on lung cancer. Some articles covered more than one type of cancer. After the screening of titles and abstracts and the reading of potentially eligible full texts, 41 reviews were included in the qualitative synthesis. Of these 41 reviews, 34 provided information about 25(OH)D levels and cancer, while the remaining 7 reviews only provided information on vitamin D intake (Table 1).

### 3.1. Breast Cancer

The results related to breast cancer (incidence and mortality) are briefly summarized in Table 2. Thirteen papers (ten SR with meta-analyses [26,27,28,29,30,31,32,33,34,35], three SR without meta-analyses [36,37,38]) were included in the umbrella review.

Four of the five reviews that analyzed the association between 25(OH)D levels and breast cancer incidence show significant inverse associations when comparing the lowest vs. highest categories of 25(OH)D with odds ratios (OR) or relative risks (RR) between 0.55 (95% CI 0.38–0.80) [26] and 0.85 (95% CI 0.74–0.98) [27]. One meta-analysis shows a significant inverse dose–response relationship (OR = 0.94 per 5 nmol/L; 95% CI 0.93–0.96) [34]. A second dose–response analysis shows a non-significant trend (OR = 0.98 per 10 nmol/L; 95% CI 0.96–1.00) [30]. The fifth meta-analysis calculates non-significant trends (RR = 0.92, 95% CI 0.83–1.02 for highest vs. lowest 25(OH)D categories; RR = 0.98, 95% CI 0.69–1.00 per 10 ng/mL increase in 25(OH)D [30]). These results suggest a protective effect of higher 25(OH)D levels. However, no statement on causality can be made, as the reviews only included observational studies.

For breast cancer mortality and survival, the results show a similar picture. With regard to 25(OH)D status, all six meta-analyses show significant inverse associations between 25(OH)D level and breast cancer mortality or survival [29,30,31,32,33,35]. The hazard ratios (HR) for overall survival are estimated between 0.56 (95% CI 0.4–0.7) [32] and 0.67 (95% CI 0.56–0.79) [29] for the comparison between the highest and the lowest 25(OH)D level. The three reviews without meta-analyses include studies with significant inverse associations [37] or report inconclusive (inverse associations and no associations) results [36,38]. These results suggest a protective role of higher 25(OH)D levels. Based on the observational studies in the reviews, no statement on causality is possible here.

### 3.2. Prostate Cancer

The results related to breast cancer (incidence and mortality) are briefly summarized in Table 3. Nine papers (five SR with meta-analyses [39,40,41,42,43], four SR without meta-analyses [31,36,37,38]) were included in the umbrella review.

Two of the four reviews that analyzed the association between 25(OH)D levels and prostate cancer incidence show significant positive associations, either when comparing the lowest vs. highest categories of 25(OH)D (RR = 1.15; 95% CI 1.06–1.24 [40] and OR = 1.17; 95% CI 1.05–1.30 [42]), or the dose–response relationship [40,42]. This indicates a chance of a higher risk for pancreatic cancer for persons with higher 25(OH)D levels. The two other, and older, reviews do not show associations between 25(OH)D levels and prostate cancer incidence [39,41].

Regarding prostate cancer mortality, three of the four reviews without meta-analysis describe a trend towards a protective influence of higher 25(OH)D levels [31,36,37]. Both meta-analyses present significant inverse associations and indicate a protective influence of higher 25(OH)D levels. Song et al. calculate significant dose–response relationships for prostate cancer specific mortality (HR = 0.91 per 20 nmol/L; 95% CI 0.87–0.97) and overall mortality of prostate cancer patients (HR = 0.91 per 20 nmol/L; 95% CI 0.84–0.99) [43]. Buttigliero et al. included just one study which calculated an adjusted hazard ratio of 0.16 (95% CI 0.05–0.43) for the highest vs. lowest 25(OH)D level [36]. However, no statement on causality can be made, as the reviews only included (a small number of) observational studies.

### 3.3. Pancreatic Cancer

The results for pancreatic cancer (incidence and mortality) are briefly summarized in Table 4. Two papers (with meta-analyses [44,45]) were included in the umbrella review.

Both reviews that studied the association between 25(OH)D levels and pancreatic cancer showed no associations [44,45].

Only one study about the association between 25(OH)D level and pancreatic cancer mortality met the inclusion criteria. The meta-analysis of Zhang et al. [45] reported an significant inverse association between 25(OH)D level and pancreatic cancer mortality (HR = 0.81; 95% CI 0.68–0.96).

### 3.4. Colorectal Cancer

The results related to colorectal cancer (incidence and mortality) are briefly summarized in Table 5. Twelve papers (nine SR with meta-analyses [31,46,47,48,49,50,51,52,53]; three SR without meta-analyses [36,37,38]) were included in the umbrella review.

All four reviews that analyzed the association between 25(OH)D levels and colorectal cancer incidence show significant inverse associations, either when comparing the lowest vs. highest categories of 25(OH)D, or the dose–response relationship [46,47,48,49]. When comparing the lowest vs. highest categories of 25(OH)D, the included meta-analyses computed an RR between 0.62 (95% CI 0.56–0.70) [46] and 0.68 (95% CI 0.60–0.78) [47]. These results suggest a protective role of higher 25(OH)D levels on colorectal cancer incidence. However, no statement on causality can be made, as the reviews only included observational studies.

Regarding colorectal cancer mortality, the results show a trend towards a positive influence of 25(OH)D. The results of all six meta-analyses [31,47,50,51,52,53] show significant inverse associations between 25(OH)D level and colorectal cancer specific mortality (HR between 0.64 (95% CI 0.56–0.73) [47] and 0.67 (95% CI 0.57–0.78) [50]), and also overall mortality of colorectal cancer patients (HR between 0.63 (95% CI 0.5–0.8) [51] and 0.69 (95% CI 0.61–0.78) [47]). The three reviews without meta-analyses include studies with significant inverse associations [36] or report inconclusive (inverse associations and no associations) results [37,38]. Again, no statement on causality can be made, as the reviews only included observational studies.

### 3.5. Lung Cancer

The results related to lung cancer (incidence and mortality) are briefly summarized in Table 6. Ten papers (seven SR with meta-analyses [31,54,55,56,57,58,59]; three SR without meta-analyses [36,37,38]) were included in the umbrella review.

Three of the five reviews with meta-analysis about the association between 25(OH)D level and lung cancer incidence show significant inverse associations, either when comparing the lowest vs. highest categories of 25(OH)D (RR = 0.84; 95% CI 0.74–0.95 [55]) or the dose–response relationship [54,55]. One review shows a significant association after sensitivity analysis and the exclusion of one study [56], and the fifth review shows no association [57]. Zhang et al. [58] draw their attention to vitamin D status (25(OH)D level plus vitamin D intake) and confirm a possible protective influence of vitamin D.

Regarding lung cancer mortality and 25(OH)D status, the results of all seven reviews are inconsistent. There are only significant inverse associations for 25(OH)D level and lung cancer specific mortality (RR = 0.76 (95% CI 0.61–0.94) [55] and OR = 0.39 (95% CI 0.28–0.54) [56]).

### 3.6. Methodological Quality

The rating of the methodological quality of the reviews that met the inclusion criteria did show shortcomings in critical domains of the AMSTAR 2 tool for most of the reviews. No review was rated as “high” quality, five were rated as “moderate” quality [29,44,45,54], and five were rated “low” [40,43,46,56,59]. The vast majority was rated as “critically low” [26,27,28,30,31,32,33,34,35,36,37,38,39,41,42,47,48,49,50,52,53,55,57,58].

## 4. Discussion

This umbrella review provides a comprehensive update on the research on vitamin D and five of the most important cancers. The use of the umbrella review approach is justified by the width of the research field and the large number of reviews on vitamin D and cancer [60,61,62]. The results of this review show inverse correlations between 25(OH)D levels and the incidence of the cancers studied, except for prostate cancer. The picture for 25(OH)D level and mortality is even more consistent. The included meta-analyses show inverse correlations for all the included cancer types.

Several limitations must be noted. *First*, it is not possible to make statements on causality because most of the studies included in the various reviews are observational studies. This leaves room for the possibility of reverse causation as criticized by Autier et al. [63] or Reijven and Soeters [64]. *Second*, there are large differences between the reviews with regard to the categorization of 25(OH)D levels; some studies work with the comparison of the highest versus the lowest category (and also differ in this respect), while others calculate dose–response relationships. It is therefore difficult to quantify the relationships described in the various publications or to specify target values. *Third*, it is important to mention that most of the reviews in this umbrella review include observational studies. This is criticized by several authors because of the wide range of epidemiological methods, different adjustment of confounders, etc. [65,66]. These problems hamper this umbrella review as well. Across all of the cancers studied arise similar problems regarding the comparability of the reviews that met the inclusion criteria. The most important challenge is that different study designs might lead to different outcomes as the blood samples are drawn at different times. In cohort studies, the blood samples are drawn prior to the diagnosis; conversely, in case control studies, the samples are drawn after the diagnosis. Therefore, it is possible that a low 25(OH)D level is a consequence of the cancer disease and not the cause of the cancer. This is discussed by several of the reviews [27,32,38,52]. Conversely, there are lengthy follow-up times in cohort studies or nested case-control studies between the blood draw and the cancer diagnosis (e.g., up to 20 years in the study by Albanes et al. [67] on prostate cancer). This issue results in a significant dearth of data regarding the 25(OH)D status during preclinical cancer development. To address this lack of data, Grant [68] recommends regular measurement of circulating vitamin D every two years for cohort studies. *Fourth*, the AMSTAR 2 [24] ratings of the reviews that met the inclusion criteria are mostly “low” or “critically low”. At first sight, this reduces the credibility of the reviews (and the credibility of the whole umbrella review). However, there are two points that need to be clarified with regard to the assessment of the reviews: firstly, it should be noted that the results of the different reviews point in a consistent direction despite the weak ratings. Secondly, it should also be noted that the AMSTAR 2 rating was only published in 2017 [24], so the older publications could not include these criteria (the same applies to “study registration” (domain 2), where corresponding databases (e.g., PROSPERO) only became established in the course of the 2010s). Authors such as Sluyter et al. [69] confirm similar experiences with the use of the AMSTAR 2 tool in the preparation of reviews of reviews. A *fifth* limitation is linked to the fourth: older reviews are notably lacking in reporting quality. Some reviews address different questions and types of cancer (a prime example is Buttigliero et al. [36]). This results in brief discussion sections in the corresponding journal articles, with insufficient attention paid to key issues such as publication bias or heterogeneity. Some review authors, (e.g., Rose et al. [33] or Maalmi et al. [50]), also note that the reporting quality of the individual studies is inadequate. *Sixth*, the umbrella review might have missed some relevant reviews as the inclusion criteria did not make specifications on nested case control studies, which are case control studies by name but follow a prospective approach [70].

Adequate 25(OH)D levels appear to be an important component in preventing cancer and improving cancer survival. However, it should also be noted that observational studies on the relationship between vitamin D supplementation and cancer have so far shown mostly inconsistent results (e.g., (subgroup) analyses in the reviews by Estebanez et al. [27], Hossain et al. [28], Kanellopoulou [71] (for breast cancer), Buttigliero et al. [36], Petrou et al. [72], Shahvazi et al. [73] (for prostate cancer), Heine-Bröring et al. [74], Huang et al. [47], Liu et al. [75], Touvier et al. [49], Vaughan-Shaw et al. [76] (for colorectal cancer)). These results are likely due to the fact that vitamin D intake via food or supplements is less important compared to cutaneous vitamin D synthesis. In addition, methodological problems, such as the inaccurate measurement of vitamin D intake in observational studies, have also contributed to these inconsistent results. For these reasons, there is a need for more and better designed studies in this area of research.

## 5. Conclusions

Adequate 25(OH)D levels and prevention of 25(OH)D deficiency (25(OH)D levels below 75 nmol/L or 30 ng/mL [77,78]) are important components of health. For most cancers studied, adequate 25(OH)D levels imply a protective influence on incidence, and even more so on mortality, whether directly or as a proxy. However, several methodological challenges make it difficult to compare the different reviews that met the inclusion criteria. It is also not possible to prove causality due to the large number of observational studies included in the underlying reviews. Therefore, further research is necessary.

## Figures and Tables

**Table 1:** Literature extraction.

	Breast Cancer	Prostate Cancer	Pancreatic Cancer	Colorectal Cancer	Lung Cancer
articles for screening (after duplicates removed)	90	52	18	69	17
retrieved full texts (after screening)	40	18	5	40	11
included in qualitative synthesis	14	11	3	15	10
**reports on 25(OH)D level**	**13**	**9**	**2**	**12**	**10**
- SR with meta-analysis (MA)	10	5	2	9	7
- SR without MA	3	4		3	3

**Table 2:** Result summary of 25(OH)D status and breast cancer.

Incidence	Mortality
5 reviews [26,27,28,30,34] (5 with meta-analyses)	9 reviews [29,30,31,32,33,35,36,37,38] (6 with meta-analyses)
4 meta-analyses [26,27,28,34] show significant inverse associations 1 meta-analysis [30] shows no significant association	6 meta-analyses [29,30,31,32,33,35] show significant inverse associations 1 review [37] reports inverse associations 2 reviews [36,38] report inconclusive results (some studies with inverse associations, some without associations included)

**Table 3:** Result summary of 25(OH)D status and prostate cancer.

Incidence	Mortality
4 reviews [39,40,41,42] (4 with meta-analyses)	5 reviews [31,36,37,38,43] (1 with meta-analysis)
2 meta-analyses [40,42] show significant inverse associations	1 meta-analysis [43] shows significant inverse associations 3 reviews [31,36,37] report positive trends (inverse associations in the included studies)

**Table 4:** Result summary of 25(OH)D status and pancreatic cancer.

Incidence	Mortality
2 reviews [44,45] (2 with meta-analyses)	1 review [45] (1 with meta-analyses)
2 meta-analyses [44,45] report no association	1 meta-analysis [45] shows significant inverse association

**Table 5:** Result summary of 25(OH)D status and colorectal cancer.

Incidence	Mortality
4 reviews [46,47,48,49] (4 with meta-analyses)	9 reviews [31,36,37,38,47,50,51,52,53] (6 with meta-analyses)
4 meta-analyses [46,47,48,49] show significant inverse associations	6 meta-analyses [31,47,50,51,52,53] show significant inverse associations 1 review [36] reports inverse associations 2 reviews [37,38] report inconclusive results (some studies with inverse associations, some without associations included)

**Table 6:** Result summary of 25(OH)D status and lung cancer.

Incidence	Mortality
5 reviews [54,55,56,57,58] (5 with meta-analyses)	7 reviews [31,36,37,38,55,56,59] (4 with meta-analyses)
3 meta-analyses [54,55,58] show significant inverse associations, 1 meta-analysis [56] with inconsistent results	all 7 reviews [31,36,37,38,55,56,59] with inconsistent results
